# Personal and social patterns predict influenza vaccination decision

**DOI:** 10.1186/s12889-020-8327-3

**Published:** 2020-02-12

**Authors:** Adir Shaham, Gabriel Chodick, Varda Shalev, Dan Yamin

**Affiliations:** 1 Department of Industrial Engineering, Tel Aviv University, 55 Haim Levanon St, Tel Aviv, Israel; 2 MaccabiTech Institute of Research and Innovation, 4 Kaufmann St, Tel Aviv, Israel

**Keywords:** Influenza, Influenza vaccination, Vaccination coverage, Vaccine refusal, Vaccination behavior, Prediction

## Abstract

**Background:**

Seasonal influenza vaccination coverage remains suboptimal in most developed countries, despite longstanding recommendations of public health organizations. The individual’s decision regarding vaccination is located at the core of non-adherence. We analyzed large-scale data to identify personal and social behavioral patterns for influenza vaccination uptake, and develop a model to predict vaccination decision of individuals in an upcoming influenza season.

**Methods:**

We analyzed primary data from the electronic medical records of a retrospective cohort of 250,000 individuals between the years 2007 and 2017, collected from 137 clinics. Individuals were randomly sampled from the database of Maccabi Healthcare Services. Maccabi’s clients are representative of the Israeli population, reflect all demographic, ethnic, and socioeconomic groups and levels. We used several machine-learning models to predict whether a patient would get vaccinated in the future. Models’ performance was evaluated based on the area under the ROC curve.

**Results:**

The vaccination decision of an individual can be explained in two dimensions, Personal and social. The personal dimension is strongly shaped by a “default” behavior, such as vaccination timing in previous seasons and general health consumption, but can also be affected by temporal factors such as respiratory illness in the prior year.

In the social dimension, a patient is more likely to become vaccinated in a given season if at least one member of his family also became vaccinated in the same season. Vaccination uptake was highly assertive with age, socioeconomic score, and geographic location. An XGBoost-based predictive model achieved an ROC-AUC score of 0.91 with accuracy and recall rates of 90% on the test set. Prediction relied mainly on the patient’s individual and household vaccination status in the past, age, number of encounters with the healthcare system, number of prescribed medications, and indicators of chronic illnesses.

**Conclusions:**

Our ability to make an excellent prediction of the patient’s decision sets a major step toward personalized influenza vaccination campaigns, and will help shape the next generation of targeted vaccination efforts.

## Background

Influenza continues to constitute a major health threat with a significant economic burden. In the United States alone, influenza is responsible for over 100,000 hospitalizations and over 4000 deaths each year [1–4]. Similar outcomes are observed in most developed countries, including in Israel [5, 6]. The most efficient way for an individual to prevent influenza infection and its complications is vaccination [7]. Because influenza is an infectious disease, vaccination also reduces the transmission of influenza, providing benefits for both the vaccinated individual and the rest of the population [8]. Long-standing CDC recommendations suggest that all individuals above six months of age [9] should be vaccinated against seasonal influenza. However, the majority of the US population does not comply with these recommendations, and vaccination rates against seasonal influenza hover around 40% annually [10]. Similar trends are observed in almost all developed countries [11]. Because the elderly above 65 are at higher risk of developing complications due to influenza, the World Health Assembly has set a less ambitious goal of 75% vaccination coverage for this specific population [12]. To encourage this goal, the OECD defined the vaccination coverage for the elderly as an important healthcare quality indicator. Nevertheless, the vast majority of developed countries including Israel fail to meet this goal.

In Israel, the national guidelines by the Ministry of Health recommend influenza vaccination for all individuals above six months of age [13] with the focus on the following groups: individuals above 65, pregnant women, healthcare workers, and individuals with certain health conditions. The Ministry of Health also recommends prioritizing individuals that were previously hospitalized due to influenza or pneumonia [14] following recent studies in Israel that indicated these populations are at elevated risk to become infected and complicated with influenza [15], as well as transmit the disease to others [16]. Since 2008, vaccination has been fully subsidized and is accessible in over 2000 health clinics located throughout the entire country. The vaccine is typically available each year between October and March, which corresponds to 1–2 months prior to the influenza season and 1–2 months after the pick of the season. The inventory in the clinics is backed-up with logistic support from the Health Maintenance Organization’s (HMO) main branches on a daily basis.

The dissonance between the importance of influenza vaccination and the observed vaccination rates suggests that more research is required to understand the individual decision of whether or not to become vaccinated. Health behavior models, such as the Health Belief Model [17], place subjective and psychological elements at the core of the individual’s health behaviors. According to these models, the decision to vaccinate is often based on personal beliefs regarding vaccinations as well as an individual’s risk perceptions, which reflect the perceived severity of influenza, an individual’s perceived susceptibility, the perceived vaccination efficiency, and the effects of worries and regrets [18–20]. Studies that rely on these models have two main methodological limitations: a) they are mostly based on small-scale and relatively homogeneous samples, and b) they were primarily derived from self-reported and subjective measurements that may lead to inherent biases. Furthermore, most of these studies did not take into account personal characteristics and behavioral patterns to predict future vaccination behavior.

Other studies in the field have analyzed large-scale data from social media and other sources. While some of these studies have focused on forecasting cases of illness [21], others aimed to capture the geographic and demographic patterns of influenza vaccination behavior relying on aggregated, rather than personal, vaccination data [22]. Although electronic medical records (EMRs) are increasingly used in research [23], even for predictive modeling [24, 25], the usage of EMRs for influenza vaccination behavior remains limited. We sought to take advantage of EMRs and demographic data at a large scale to address this gap in the literature. To our knowledge, this is the first study to analyze and predict the decisions of individuals to get vaccinated against influenza using personal, detailed, and longitudinal data. The goals of this study were to: a) Identify personal and social behavioral patterns and indicators for influenza vaccination uptake, and b) use these indicators to develop a machine-learning model that would predict vaccination decisions of individuals in the upcoming influenza season.

## Methods

### Data description and case definition

We analyzed primary data from anonymized electronic medical records of 250,000 individuals between the years 2007 and 2017, collected from Maccabi Healthcare Services (Maccabi). Maccabi is the second-largest health maintenance organization (HMO) in Israel, serving about 25% of the population (2,215,000 clients). Maccabi’s clients are representative of the Israeli population, and reflect all demographic, ethnic, and socioeconomic groups and levels [26].

In order to avoid biases that may arise from death or changes in healthcare provider, we randomly selected 250,000 individuals who were members of Maccabi during the entire period from 2007 to 2017 or who were born during this period and remained members until 2017. We chose 2008 as the earliest influenza season because it was the first season that the vaccination was offered free of charge for all members of all the health-care providers in Israel [27].

For each member, we compiled demographic characteristics, influenza vaccination history, respiratory diagnoses, prescriptions, encounters with the healthcare system, hospitalizations, chronic illnesses, and family connections to other members in the data set (Additional file 1: Table S1). The data were approved for use by Maccabi’s sub-Helsinki institutional review board, signed by Dr. Yosef Azuri, protocol number 0048–17-BBL.

#### Season

Influenza is a seasonal disease, with the highest prevalence between October and March [28]. Therefore, we defined each “season” as the period ranging from June 1 until May 31 of the following year and named it after the end year. For example, “season 2016” was defined as the period between June 1, 2015 and May 31, 2016.

#### Age group

Our data specifies for each member the year of birth. Thus, we divided the population into the seven age groups (0–4, 5–16, 17–25, 26–35, 36–50, 51–64, and 65+). This division was done with respect to the season in question; if a specific analysis included several seasons, the population was divided into age groups separately in each season. Therefore, in some cases, a patient belonged to one age group in a specific season and moved to another age group in the following season.

#### Respiratory illness

We define a respiratory illness as a respiratory diagnosis reported by a physician, according to several codes from the International Statistical Classification of Diseases and Related Health Problems protocol (ICD-9; Additional file 1: Table S2). Given that perceptions rather than the actual cause of infection govern the decision for an individual to get vaccinated against influenza [18–20], analyzed this considerably broad definition rather than using ICD-9 codes that are limited to influenza or influenza-like-illness.

#### Family

Family members are defined as a set of patients having the same “family code” in the surveillance systems of Maccabi. Among the 250,000 patients included in our study, 55,749 patients shared a “family code” with at least one other patient within the data, creating 25,999 families.

To accomplish the two goals of this study, we established three discrete steps. The first step was *personal pattern analysis*, in which we identified behavioral patterns that relate to the personal aspects of an individual’s vaccination decision. The second step was *social pattern analysis*, in which we evaluated the environmental factors that may influence an individual’s vaccination decision. Finally, in the *predictive modeling step*, we developed a machine learning model that converted the data and the insights from the previous tasks into an individual-level prediction of a future vaccination decision. The third step was conducted in accordance with the tripod statement [29] for multivariable prediction models.

A. Descriptive personal patterns

We examined the behavior of a random variable representing the probability of a patient to become vaccinated in a given season, as a function of the patient’s vaccination decision and respiratory illnesses in the previous season. For each season (among the 2009–2017 seasons), we divided the population into seven age groups and then divided each age group into four mutually exclusive sub-groups: 1) patients who vaccinated and became infected in the previous season, 2) patients who vaccinated and did not become infected in the previous season, 3) patients who were not vaccinated and became infected in the previous season, and 4) patients who were not vaccinated and did not become infected in the previous season. We calculated the proportion of patients who became vaccinated in the given season for each of the 28 groups.

We then created a measurement that encodes the time during the season that each patient became vaccinated, and named it the patient’s *Average Vaccination Rank*. In each season, each patient received a value within the range [0–1], representing the timing of the vaccination within the season relative to other patients. The first patient to become vaccinated received the value 1, and the last patient to become vaccinated received the value 0. The patient’s *Average Vaccination Rank* was defined as the average of these values across seasons the patient became vaccinated in. In order to examine the distribution of the *Average Vaccination Rank*s among patients, we divided the population into 11 mutually exclusive groups according to the number of vaccinations in a 10-season period. For this purpose, we excluded patients under 10 years old. We then used boxplot analysis to depict the distribution of the *Average Vaccination Rank* among each group separately by age groups.

We also examined the relationship between healthcare consumption and vaccination decision. We used two measurements for this purpose: 1) the average number of prescribed medications (of any kind) per season, and 2) the vaccination proportion, calculated as the number of seasons that the patient became vaccinated out of 10 seasons, or less for patients under 10. We then divided the population into 10 equal groups by deciles of the average number of prescriptions and examined the distribution of the vaccination proportion for each group separately using boxplot analysis. In addition, we conducted a similar analysis for the average number of encounters with the healthcare system (of any kind).

B. Descriptive Social patterns

*Family analysis* – we analyzed the vaccination decision of patients with respect to the vaccination decisions of other members of the patients’ families. We then calculated a relative risk for becoming vaccinated, defining the exposed group as the group of patients with at least one other family member who became vaccinated in a given season. This analysis was conducted for all seasons combined. We also examined the similarities between family members over time and compared it to the similarities between randomly sampled patients (Additional file 1: Figure S1).

*Geographic analysis* – Each patient was associated with one of 137 Maccabi clinics, where he receives most of his medical care. We used the vaccination proportion, calculated as the number of seasons the patient became vaccinated out of 10 seasons (or less for patients under 10) and grouped the patients by clinic. We then conducted an ANOVA test to examine the variance between clinics. In addition, we calculated the proportion of patients who became vaccinated, for each season, at each clinic. We then took all 3070 statistical areas in Israel (according to the Israeli Central Bureau of Statistics) and associated each statistical area with its nearest clinic. Using these data, we created a heat-map that displays the variance in vaccination proportion across socioeconomic areas based on the average vaccination proportion across all seasons in the associated clinics.

*Socioeconomic analysis* - We performed an analysis of the vaccination proportion with respect to the socioeconomic score, range from 1 to 10, that was assigned to each patient by Maccabi. We calculated the average vaccination proportion for each socioeconomic score across all seasons and displayed the variance of the vaccination proportion of different socioeconomic scores.

C. Predictive modeling

We developed a model to predict the influenza vaccination behavior of a patient in a future influenza season. The predictive model development was guided by and made in accordance with the tripod statement [29] for multivariable prediction models. This is a classification problem with a binary label, as the patient may or may not become vaccinated (positive label or negative label, respectively). We created a time-free model, which does not attempt to predict a patient’s behavior in a specific season, but rather allows predicting behavior in any future season given the relevant previous data. Based on the results of an entropy analysis (SI Appendix), we used the data of three consecutive seasons in order to predict the behavior in the subsequent fourth season. For example, we data from seasons 2008 through 2010 to predict each patient’s behavior in 2011, data from seasons 2009 through 2011 were used to predict behavior in 2012, and so on. Therefore, the ten-season period data of each patient produced up to seven records with seven labels (seasons 2011–2017), depending on the patient’s age, creating a dataset of 1,553,907 records in total.

The features we used for the prediction were based on demographic attributes of the patient and on calculated or aggregated medical characteristics. In order to build a comprehensive model, the features of the dataset were rather basic. Because 55,749 of the patients had family members within the data, we could extract family-related features in addition to the basic features. We therefore divided the 1,553,907 records into 2 datasets: a basic dataset containing 1,225,032 records, where each record had 27 features and a label, and a ‘family’ dataset containing 328,875 records, where each record had 30 features and a label. The features are described under *Sociodemographic & EMR* in Table 1 (a detailed description can be found in Additional file 1: Table S3).

**Table 1:** Features of the models used in this study. “V” represents that a specific feature was used in a specific model

Feature(s)	*Socio demographic* model	*Vaccination decision in the previous season* model	*Vaccination decision in the previous season & Socio demographic* model	*Socio demographic & EMR model*
Age	V		V	V
Gender	V		V	V
Socioeconomic scores	V		V	V
Country of origin				V
Year of immigration				V
Vaccination indicator in the previous season		V		
Vaccination rank in the three previous seasons			V	V
Number of respiratory diagnoses in the three previous seasons				V
Cumulative hospitalized days in the three previous seasons (hospitalizations of any reason)				V
Number of encounters with the healthcare system in the three previous seasons (encounters of any reason)				V
Number of prescribed medications in the three previous seasons (any kind of medications)				V
Chronic illness in the three previous seasons				V
Vaccination proportion at the patient’s clinic, in the three previous seasons	V		V	V
Average Vaccination rank of the patient’s family in the three previous seasons				V

We trained several models using the following algorithms: 1) Logistic Regression [30], 2) Naïve Bayes [30], 3) XGBoost Random Forest [31], 4) Light GBM Random Forest [32] and 5) Artificial Neural Network [30]. While preprocessing, we used min-max scaling where relevant. Random Forest algorithms were trained using 500 decision trees.

We split both the basic dataset and the family dataset into training sets (70%), validation sets (15%), and test sets (15%). We trained each algorithm on both train sets, creating two models, one for each dataset (basic dataset and family dataset), using 4-fold cross-validation to maximize the ROC AUC score. When training the models, we conducted hyper-parameters tuning using grid-search to discover the combination of parameters that performed best on the train set. Detailed description of the hyper-parameters tuning is presented in (Additional file 1: Table S4).

After the training phase, we received two trained models for each algorithm. We predicted the labels of the records in the validation sets and evaluated the performance of the models using the ROC AUC score, precision, recall, and F1-score.

As a benchmark, we created four additional simple models. The first is the *Age* model and it relies on the patient’s age alone. The second one is the *Sociodemographic* model (SD), which does not utilize personal medical records, but instead uses a subset of demographic features of the patient and the vaccination coverage in the patient’s clinic. The third is *Vaccination decision in the previous season* model (PS), which relies solely on the patient’s behavior in the previous season; this model predicts that an individual that became vaccinated in the last season will become vaccinated in the predicted season, and vice versa. The fourth simple model, the *Vaccination decision in the previous season & Sociodemographic* model (PS&SD), uses the patient’s behavior in the previous season in addition to the features used by SD model. The features of the models are described in Table 1. Because none of the simple models use family-based features, they were all trained over the entire training population and predicted the labels for all validation samples. The training of these simple models was based solely on the algorithm that performed best on the basic and family datasets.

Finally, we evaluated the most successful models and the simple models on the test sets, and analyzed the feature importance of these models.

## Results

Overall, we found several personal and social behavioral patterns that are associated with a patient’s tendency to become vaccinated and that provide predictive insight into the future vaccination decision of the patient. Furthermore, these patterns served as the foundations for a machine-learning modeling phase, which yielded an accurate and comprehensive predictive model for the patient’s vaccination decision in the next season.

### Descriptive personal patterns

The average seasonal vaccination coverage is approximately 20%. Of the 250,000 patients included in this study, 101,407 (41%) became vaccinated at least once, 32,584 (13%) became vaccinated at least five times, and only 7609 (3%) became vaccinated during this entire 10-year period. The age of the patients in 2017, ranged from 1 year to 116 years, with a median of 36 years (first quartile = 12 years, third quartile = 53 years). Fifty-two percent of the patients were female.

**Fig. 1:**
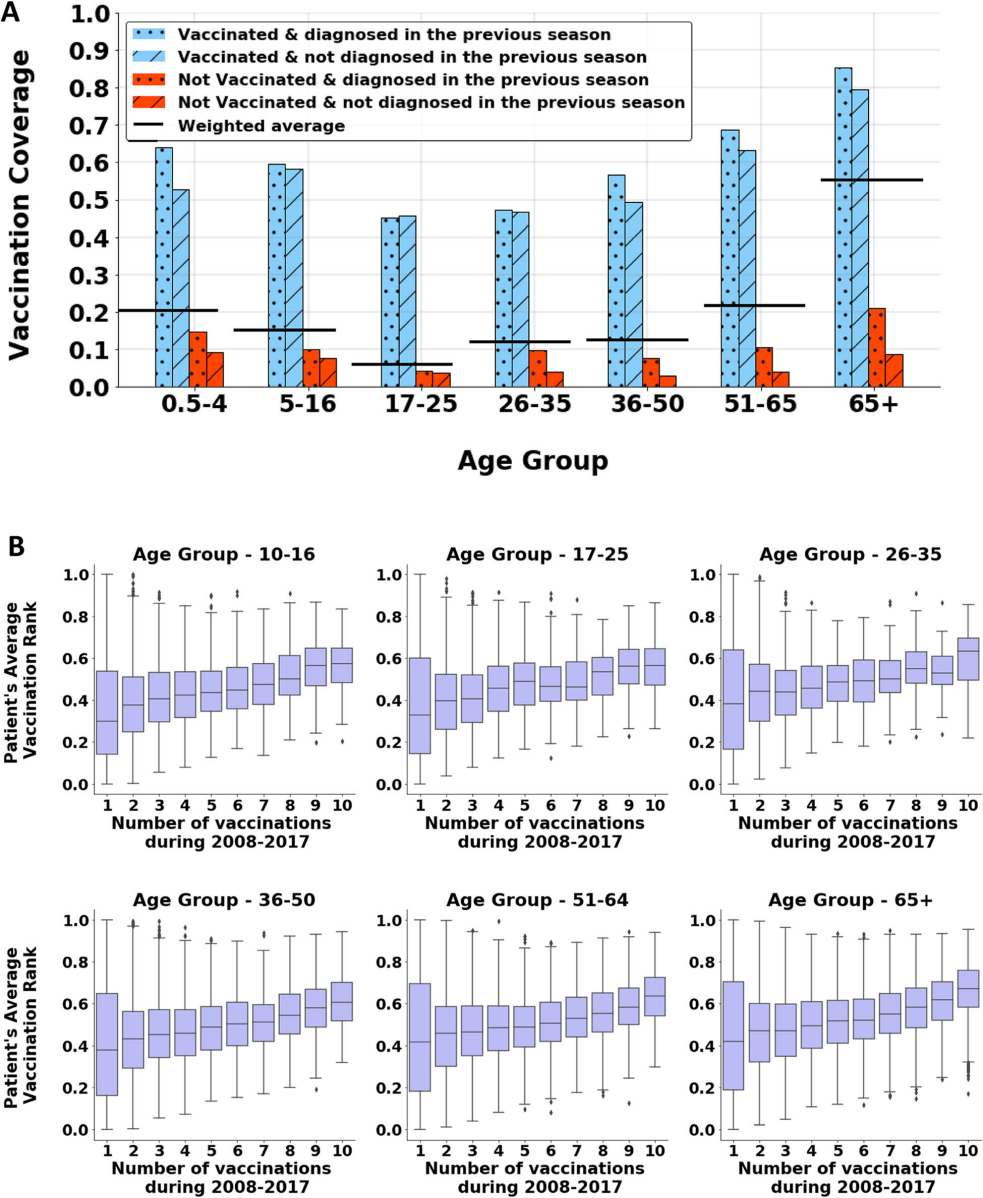
**a** Vaccination coverage in the 2017 season with respect to vaccination decision and respiratory diagnoses in the 2016 season. Because not all groups are of equal size, the horizontal black line indicates the weighted vaccination coverage of each age group. For (**a**), the confidence intervals are extremely small, and therefore not displayed, due to the large size of the groups (more than 10,000 individuals in each group). Similar patterns were observed in every pair of consecutive seasons from 2008 until 2017. **b** Boxplots of the *Average Vaccination Rank* by the number of vaccinations during the seasons 2008–2017. Every patient who became vaccinated at least once resides in exactly one boxplot. Each group consists of more than 4000 individuals. The higher the *Average Vaccination Rank*, the earlier the patient’s average vaccination timing within the season. These graphs show that an individual who becomes vaccinated more often also tends to do so at an earlier stage of the season, regardless of his or her age group

By examining the decision of a patient to become vaccinated in a given season with respect to the patient’s vaccination decision and respiratory diagnosis in the season prior, we found two main patterns: 1) The default effect – a patient is much more likely to become vaccinated if she or he has done so in the previous season; 2) Patients who were not vaccinated in the previous season and were also diagnosed with respiratory illness were more likely to become vaccinated in the next season (Fig. 1a). We further found that the more frequently a patient becomes vaccinated, the higher the patient’s *Average Vaccination Rank*, meaning that the patient tends to become vaccinated in an earlier stage of the season. These findings were consistent in all age groups (Fig. 1b).

**Fig. 2:**
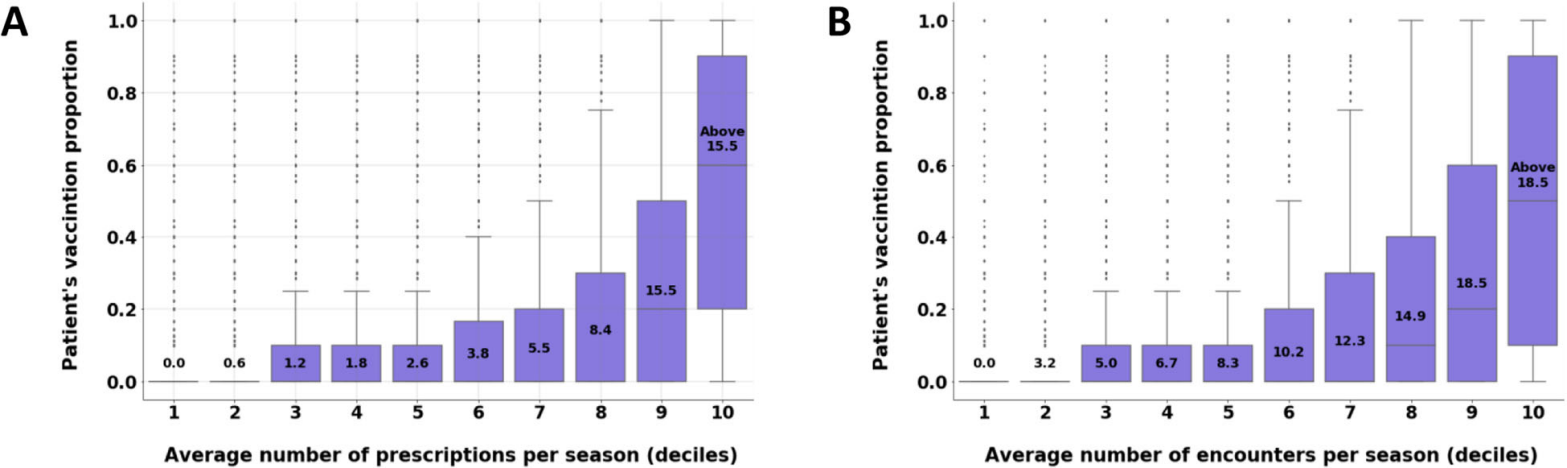
Vaccination rate and healthcare consumption. Boxplot charts of the average vaccination rank by (**a**) the number of vaccinations during seasons 2008–2017, and (**b**) the number of encounters with the healthcare system during seasons 2008–2017. The deciles values are displayed on the boxes

Finally, both the average number of prescriptions and the average number of encounters with the healthcare system are associated with vaccination proportion. Patients that scored higher on these metrics (i.e., those with more prescriptions and more interactions with the healthcare system) are also characterized by higher values of vaccination proportion (Fig. 2).

### Descriptive - social patterns

**Fig. 3:**
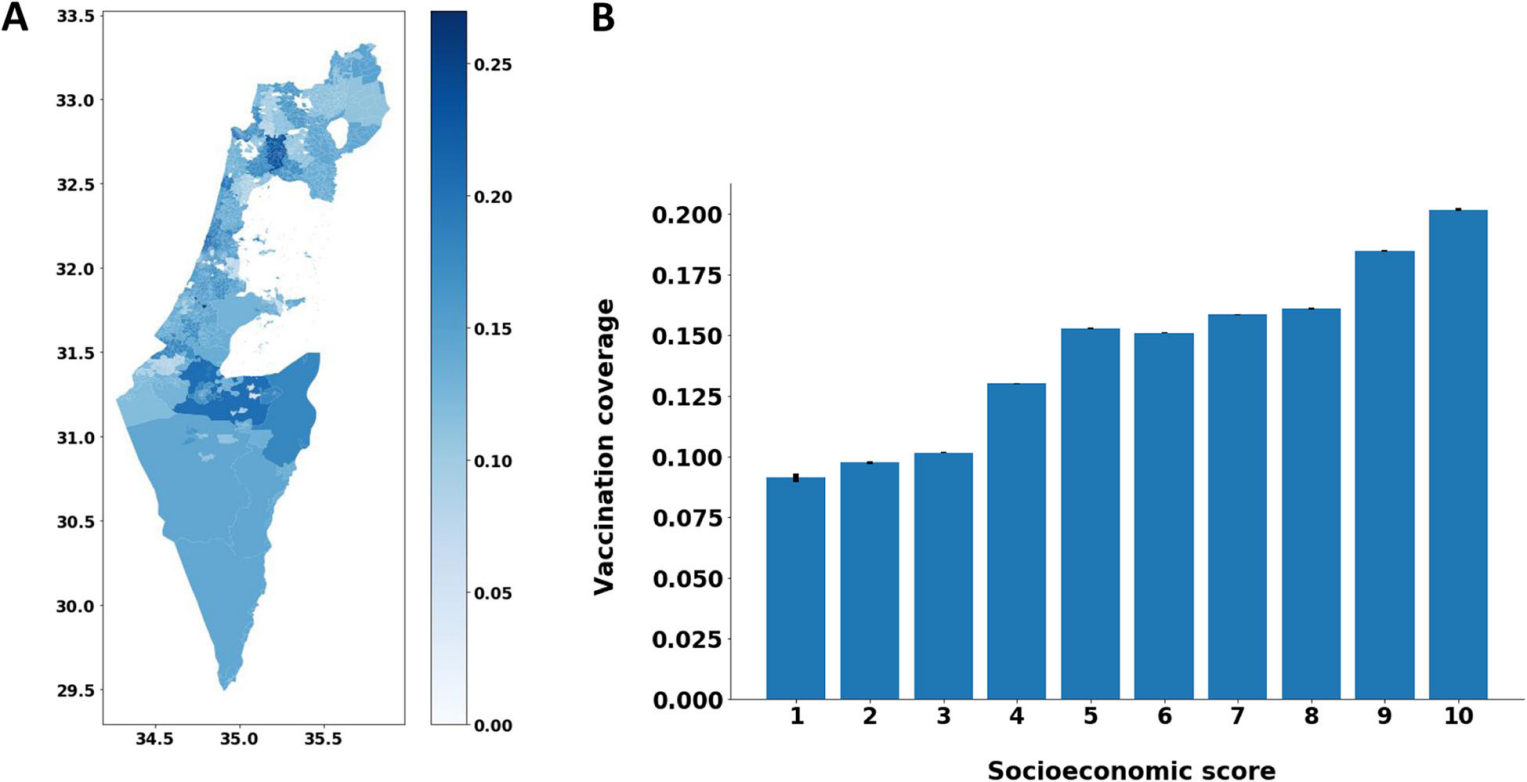
**a** Geographical heat map of the average vaccination rates across season 2008–2017. The axes represent the longitude and latitude. The darker the color, the higher the average vaccination coverage against influenza across all seasons. The map was generated using the GeoPandas open source project, http://geopandas.org/. **b** A Bar plot of the average of the vaccination proportions across all seasons as a function of socioeconomic score. The 95% confidence interval is displayed on top of each bar

By analyzing the vaccination decision of patients with respect to the vaccination decisions of their family members, we found that a patient is 11.09 times more likely to become vaccinated in a given season, if at least one member of his or her family also became vaccinated in the same season (RR: 11.09; 95% CI: 10.92 to 11.25). Our geographic analysis revealed significant differences between different clinics (*P* value <.0001), with average vaccination rates ranging between 4.4 and 25.3%. A geographical heat map of the average vaccination rates displays these differences among the statistical areas of Israel (Fig. 3a). We denote these high variations between statistical areas were observed despite similar accessibility to vaccination. In addition, we observed that a higher socioeconomic score is associated with a higher vaccination rate (Fig. 3b).

### Predictive modeling

In the basic datasets (train, validation and test), the age of the patients, as of 2017, ranged from 3 years to 116 years, with a median of 43 years (first quartile = 19 years, third quartile = 58 years). Fifty-two percent of the patients were female. In the family datasets (training, validation, and test), the age of the patients in 2017 ranged from 3 years to 94 years, with a median of 17 years (first quartile = 11 years, third quartile = 45 years). Fifty-three percent of patients in the family datasets were female.

We evaluated all models using the precision, recall, F1 score and ROC AUC measurements (Additional file 1: Table S4). The most successful models, which utilize sociodemographic and EMR data, were the XGBoost and LightGBM algorithms, with an ROC AUC score of 0.91 on the basic validation and test sets and 0.88 on the family validation and test sets. Although the XGBoost achieved slightly better F1-scores, we found that while the XGBoost holds a higher precision than the LightGBM for positive-label records (i.e., individuals who became vaccinated), its recall among these records is relatively lower. These differences may arise from the differences between the two algorithms [32], which create different decision forests and therefore different predictions.

**Fig. 4:**
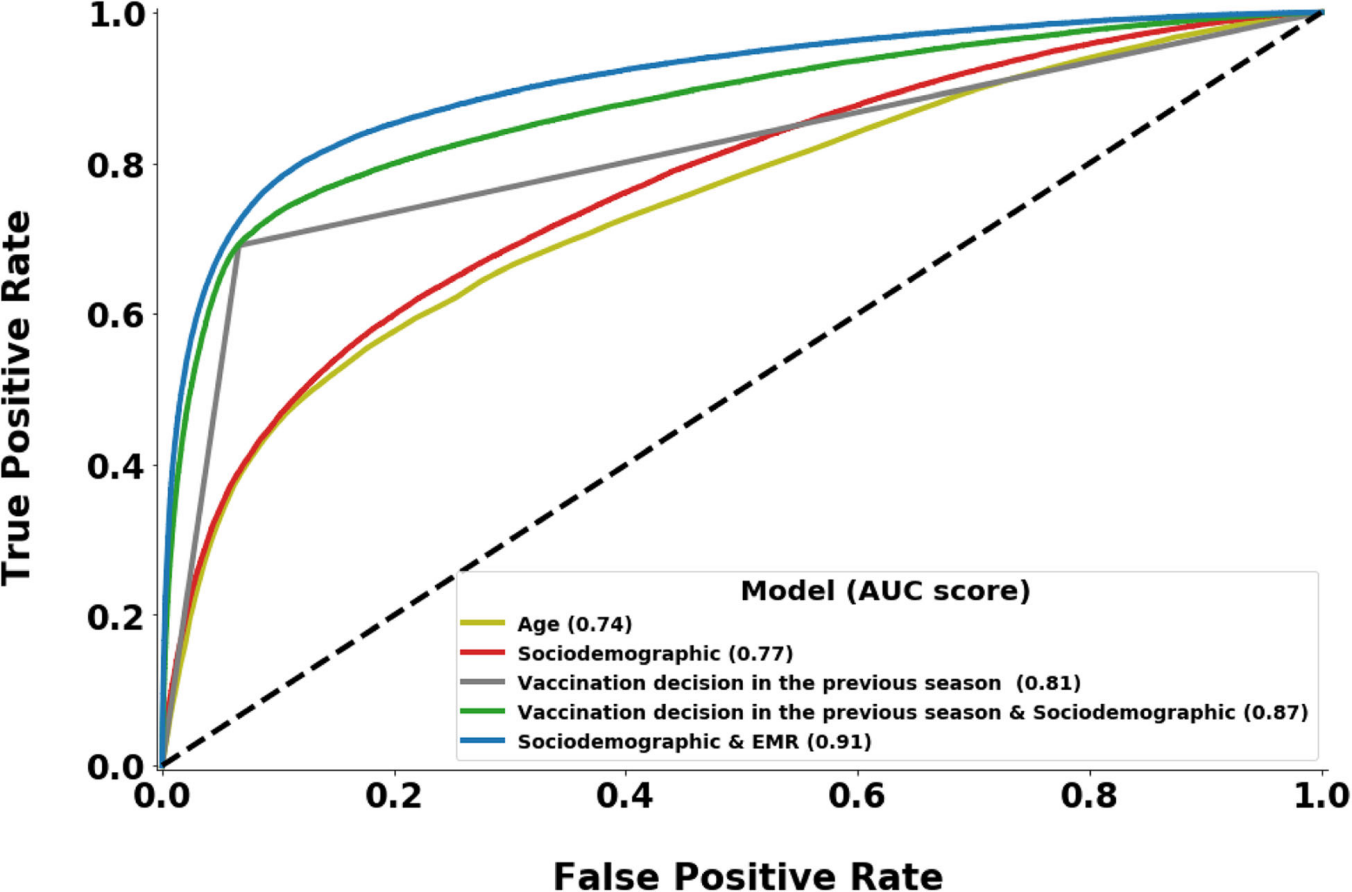
Receiver operating characteristic (ROC) curves comparison

All four simple models – *Age*, *Sociodemographic* (SD), *Vaccination decision in the previous season* (PS), and *Vaccination decision in the previous season & Sociodemographic* (PS&SD) achieved lower scores than did the XGBoost algorithm in all measurements. The PS&SD model was the most successful of the four, with an ROC AUC score of 0.87 on the validation and test sets. An ROC AUC comparison of the four simple models and the XGBoost, which was the most successful model of those utilizing *sociodemographic & EMR data*, is shown in Fig. 4.

According to a feature importance analysis for both the XGBoost and LightGBM models, the patient’s previous vaccination decisions are the most prominent features in the algorithms’ prediction process. When data were available, the vaccination decisions of a patient’s family also played a meaningful role in prediction. These features were followed by other personal features such as the patient’s age, the number of encounters with the healthcare system, the number of prescribed medications and chronic illnesses. The five most prominent features in the algorithms’ prediction process of the XGBoost model, the LightGBM model, and the simple models (except the *Age* model) are presented in Table 2. An elaborated feature importance plots is presented in Additional file 1: Figure S2.

**Table 2:** Cumulative contributions of the ranked top five features to each model

Feature’s Rank	XGB model for the basic dataset (ROC AUC score: 0.91)	XGB model for the family dataset (ROC AUC score: 0.88)	LightGBM model for basic dataset (ROC AUC score: 0.91)	LightGBM model for family dataset (ROC AUC score: 0.88)	*Socio-demographic* model (ROC AUC score: 0.77)	*Vaccination decision in the previous season & Sociodemographic* model (ROC AUC score: 0.87)
1st	Vaccination rank in the season prior (29%)	Vaccination rank in the season prior (27%)	Vaccination rank in the season prior (47%)	Vaccination rank in the season prior (30%)	Age (67%)	Vaccination rank in the season prior (68%)
2nd	Vaccination rank two seasons prior (51%)	Vaccination rank two seasons prior (39%)	Vaccination rank two seasons prior (69%)	Average Vaccination rank of the patient’s family in the season prior (51%)	Vaccination proportion at the patient’s clinic, in the season prior (74%)	Age (87%)
3rd	Vaccination rank three seasons prior (60%)	Average Vaccination rank of the patient’s family two seasons prior (45%)	Vaccination rank three seasons prior (81%)	Vaccination rank two seasons prior (60%)	Vaccination proportion at the patient’s clinic, two seasons prior (80%)	Vaccination proportion at the patient’s clinic, in the season prior (91%)
4th	Number of prescribed medications in the season prior (67%)	Average Vaccination rank of the patient’s family in the season prior (49%)	Age (85%)	Average Vaccination rank of the patient’s family two seasons prior (66%)	Socioeconomic score (1) (87%)	Vaccination proportion at the patient’s clinic, two seasons prior (93%)
5th	Chronic illness in the season prior (72%)	Chronic illness two seasons prior (53%)	Number of encounters with the healthcare system in the season prior (89%)	Age (70%)	Vaccination proportion at the patient’s clinic, three seasons prior (97%)	Vaccination proportion at the patient’s clinic, three seasons prior (96%)

## Discussion

From a large-scale analysis of electronic medical records and demographic data, we identified several behavioral patterns regarding influenza vaccination and developed a machine-learning model that predicts the decision-making of the individual in the next season. Our results indicate that the vaccination decision of an individual can be explained in two dimensions – personal and social. The personal dimension is strongly shaped by a “default” behavioral approach, which is often manifested in repeated vaccination decisions in subsequent seasons, a preferred timing of vaccination within the season. This approach is associated with a patient’s healthcare consumption measurements. We also found evidence that this “default” behavior could be affected by temporal effects such as a recent respiratory illness diagnosis, suggesting that experiencing a recent respiratory illness changes patients’ perception of the risk associated with influenza. The social dimension is divided into the social environment and immediate relatives. We observed significant differences in vaccination rates between geographical regions and patients with different socioeconomic scores. We also observed that family members tended to have similar vaccination decisions. It is likely that while the social environment sets a general approach towards influenza vaccination as a context, immediate relatives may influence the ad-hoc decision of an individual.

These patterns served as a foundation for our predictive machine learning models, which take advantage of the EMR data and demographic data to provide an excellent prediction of an individual’s future vaccination decision. Previous vaccination decisions, along with age, the number of encounters with the healthcare system, and prescriptions, serve as the strongest predictors of a future vaccination decision. In cases where the family vaccination decision in previous seasons is provided, it also serves as a strong predictor. The simple models demonstrate how a considerably accurate prediction is achieved even when only a small subset of the patient’s personal and social information is available.

The behavioral patterns that we observed by analyzing the Israeli population are consistent with widely held hypotheses regarding individual decision making in several key ways. First, our work corroborates the importance of previous vaccination decisions and the influence of an individual’s social circle in predicting future decisions [20, 22]. However, our study extends these behavioral concepts by using large-scale longitudinal data and objective measurements. Despite the inherent differences between cultures and healthcare systems worldwide, we believe that the behavioral patterns and the predictive models we have developed can be reproduced with minor modifications in most developed countries.

This study identifies and relies on correlations and associations, in both pattern analysis and predictive modeling, and does not attempt to assume or imply causality. In addition, this study does not explicitly account for intervention efforts by Maccabi during the study period or any possible effects of media exposure. The collected data do not contain information on vaccination programs in workplaces, which are relatively effective in Israel [33], and yet small-scale. For privacy, our data include for each member, only the year of birth. This result in a small bias, because patients under six month are not eligible for vaccination. However, we expect this bias to be minor, not only as this age group accounts for < 0.1% of the population but also as vaccination season lasts for 3–6 months.

Despite these limitations, and because most individuals do not become vaccinated on a seasonal basis, our ability to make an excellent prediction of a patient’s decision-making represents a major step towards personalized influenza vaccination campaigns. In this way, our work can help shape the next generation of targeted and customized vaccination efforts. Our study demonstrates how personal EMRs and demographic data can give new insight into a patient’s perceptions and can serve as a platform to anticipate the most likely decision of an individual. This predictive capacity will likely be valuable for healthcare providers and health insurance agencies who may wish to design intervention efforts for their patients or to assess the required number of doses of influenza vaccination.

## Conclusion

Influenza vaccination decision of an individual is highly predictable and can be identified even in the absence of detailed medical information about the patient. Predictors primarily include vaccination timing in previous seasons and general health consumption, but can also be affected by temporal factors such as respiratory illness in the prior year. Other predictors include vaccination uptake of family members and vaccination coverage in the individual’s main clinic in the year prior, as well as standard socio-demographic characteristics. Our ability to make an excellent prediction of the patient’s decision sets a major step toward personalized influenza vaccination campaigns against vaccine refusales, and will help shape the next generation of targeted vaccination efforts.

## Supplementary information

**Additional file 1: Table S1.** Description of the data set. **Table S2.** ICD-9 codes for the case definition of respiratory illness. **Table S3.** Detailed description of the models’ features. **Table S4.** Hyper-parameters for the machine learning models. A detailed evaluation of all predictive models. **Figure S1.** Entropy analysis of the probability to become vaccinated in 2017.The black dot represents the level of entropy with no information, the red dot represents the level of entropy when age groups distribution is provided, and the blue dots represent the levels of entropy when age group distribution is provided with increasing (left to right) amount of historical influenza-vaccination decisions data. Similar results were observed for all seasons between 2012 and 2017. **Figure S2.** Feature importance plots for the following models. **(**A) XGBoost for the basic dataset (B) LightGBM for the basic dataset (C) XGBoost for the family dataset (D) LightGBM for the family dataset (E) Sociodemographic model (F) Vaccination decision in the previous season & Sociodemographic model. X-axis values represent the total percentage of information that was gained by the splits of the feature in all the decision trees of the random forest. Y-axis values represent the features’ indices according Table S3.

## Data Availability

The data that support the findings of this study are available from the Maccabi Health Services but restrictions apply to the availability of these data, which were used under license for the current study and so are not publicly available. Data are however available from the authors upon reasonable request and with permission of Maccabi Health Services.

## Abbreviations

AUC: Area under the Curve
EMR: Electronic Medical Record
ROC: Receiver operating characteristic
RR: Relative risk
