# Supplementary material for: Personal and social patterns predict influenza vaccination decision
Source: BMC Public Health. 2020 Feb 12;20:222. doi: 10.1186/s12889-020-8327-3 (PMC7017468; doi:10.1186/s12889-020-8327-3)
Supplement: Supplementary file 1 — Additional file 1: Table S1. Description of the data set. Table S2. ICD-9 codes for the case definition of respiratory illness. Table S3. Detailed description of the models’ features. Table S4. Hyper-parameters for the machine learning models. A detailed evaluation of all predictive models. Figure S1. Entropy analysis of the probability to become vaccinated in 2017.The black dot represents the level of entropy with no information, the red dot represents the level of entropy when age groups distribution is provided, and the blue dots represent the levels of entropy when age group distribution is provided with increasing (left to right) amount of historical influenza-vaccination decisions data. Similar results were observed for all seasons between 2012 and 2017. Figure S2. Feature importance plots for the following models. (A) XGBoost for the basic dataset (B) LightGBM for the basic dataset (C) XGBoost for the family dataset (D) LightGBM for the family dataset (E) Sociodemographic model (F) Vaccination decision in the previous season & Sociodemographic model. X-axis values represent the total percentage of information that was gained by the splits of the feature in all the decision trees of the random forest. Y-axis values represent the features’ indices according Table S3. [file 12889_2020_8327_MOESM1_ESM.docx]

**SI Appendix**

**Table S1.** Description of the data set

| # | Main content | No. of records | Fields | Comments |
| --- | --- | --- | --- | --- |
|  | Demography | 250,000 | Patient anonymized ID  Registration date  Year of birth  Gender  Personal general physician anonymized ID  Geographic area  Socioeconomic score (1)  Socioeconomic score (2)  Country of origin  Date of immigration | The area name matches the location of the HMO branch of which the patient belongs.  Socioeconomic score (1) was determined by the Israeli Central Bureau of Statistics.  Socioeconomic score (2) was determined by Points Business Mapping Ltd |
|  | Vaccinations | 472,019 | Patient anonymized ID  Vaccination date |  |
|  | Respiratory diagnoses | 1,994,034 | Patient anonymized ID  Diagnosis date  Diagnosis ICD-9 code | For the list of all relevant ICD-9 codes, see Table S2 |
|  | Prescriptions | 36,916,227 | Patient anonymized ID  Prescription serial number  Medicine generic code  Medicine generic name  Date of writing  Physician anonymized ID  Number of prescribed doses  Number of days for medical treatment |  |
|  | Encounters with the healthcare system | 35,317,308 | Patient anonymized ID  Date of visit  Care type | Care type is a medical specialty or and subspecialty, e.g. cardiology, neurology. |
|  | Hospitalizations | 264,343 | Patient anonymized ID  Start date  End date  Hospital department |  |
|  | Chronic illness | 250,000 | Patient anonymized ID  Diabetes diagnosis date  Diabetes type  CVD diagnosis date  TIA diagnosis date  PVD diagnosis date  CVA diagnosis date  Cancer diagnosis date  Transplant date  Obesity diagnosis date  Osteoporosis diagnosis date  COPD diagnosis date  CKD diagnosis date  CKD stage | In most cases, where a patient was not diagnosed with a specific chronic illness, the value was NULL. |
|  | Families | 55,749 | Patient anonymized ID  Head-of-family anonymized ID | This table groups 55,749 patients into 25,999 families with at least 2 members, based on a family code.  We assume that most of the families are missing some members because the data were sampled without any respect to the “family code” of the patients. |

**Table S2.** ICD-9 codes for the case definition of respiratory illness

| # | ICD-9 Code | Description |
| --- | --- | --- |
|  | 465.9 | Acute upper respiratory infections of unspecified site |
|  | 478.8 | Upper respiratory tract hypersensitivity reaction, site unspecified |
|  | 478.9 | Other and unspecified diseases of the upper respiratory tract |
|  | 480.0 | Viral pneumonia |
|  | 480.9 | Viral pneumonia, unspecified |
|  | 481.0 | Pneumococcal pneumonia |
|  | 482.3 | Pneumonia due to Streptococcus |
|  | 483.0 | Mycoplasma pneumonia |
|  | 485.9 | Bronchopneumonia |
|  | 486.0 | Pneumonia, unspecified organism |
|  | 486.9 | Bacterial pneumonia |
|  | 487.0 | Influenza H1N1 |
|  | 487.1 | Influenza with other respiratory manifestations |
|  | 487.8 | Influenza with other manifestations |
|  | 507.0 | Aspiration pneumonia |
|  | 516.8 | Other specified alveolar and parietoalveolar pneumonopathies |
|  | 518.3 | Eosinophilic pneumonia |
|  | 780.7 | Post-influenzal asthenia |
|  | V04.8 | Vaccination-need influenza |

**Entropy analysis**

In order to determine the amount of historical data needed for future prediction, we conducted an entropy analysis of the random variable representing the probability to become vaccinated in a given season as a function of the vaccination decisions in the previous season and the patient’s age group. For each season, among the seasons 2012-2017, we calculated the entropy of the random variable in several conditions, characterized by the provided information: 1) no information provided, 2) age group distribution, 3) age group distribution and vaccination decision in the previous season, 4) age group distribution and vaccination decisions in the two previous seasons, 5) age group distribution and vaccination decisions in the three previous seasons, 6) age group distributions and vaccination decisions in the four previous seasons.

We found that the entropy of this random variable decreases slightly when age group distribution is provided and decreases significantly when vaccination decision in the previous season is provided. We also found a diminishing marginal contribution as more historical information regarding vaccination decisions is taken into account (figure S1).

| 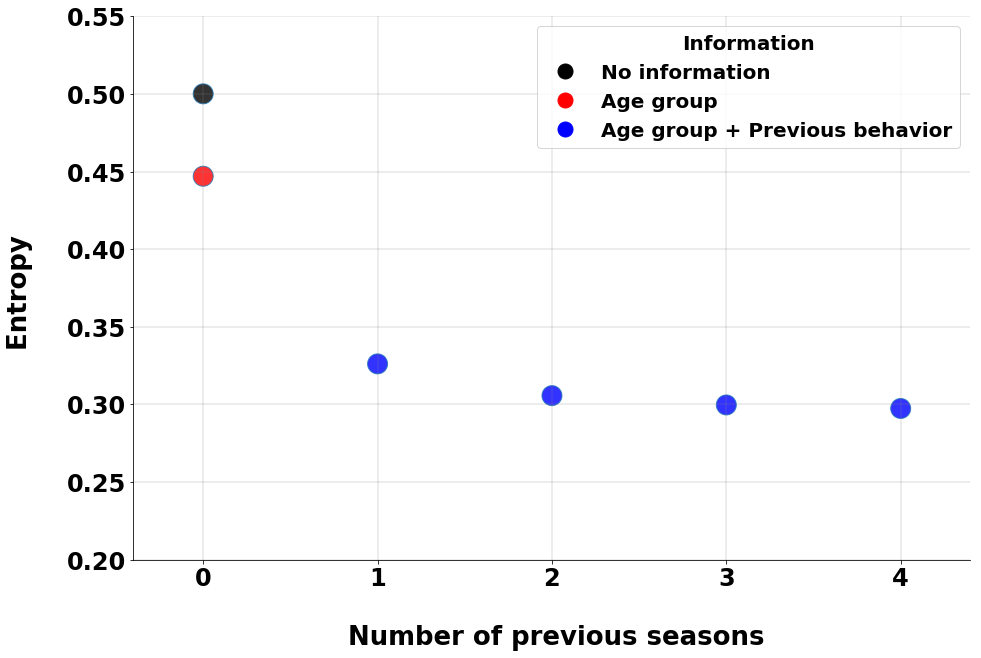 |
| --- |
| **Figure S1 – Entropy analysis of the probability to become vaccinated in 2017.** The black dot represents the level of entropy with no information, the red dot represents the level of entropy when age groups distribution is provided, and the blue dots represent the levels of entropy when age group distribution is provided with increasing (left to right) amount of historical influenza-vaccination decisions data. Similar results were observed for all seasons between 2012 and 2017. |

**Table S3.** Detailed description of the models’ features

| # | Feature | Type | Description and notes | *Socio-demographic* model | *Vaccination decision in the previous season & Sociodemographic* model |
| --- | --- | --- | --- | --- | --- |
|  | Age | Numerical |  | V | V |
|  | Gender | Binary |  | V | V |
|  | Socioeconomic score (1) | Numerical | Socioeconomic score (1) was determined by the Israeli Central Bureau of Statistics | V | V |
|  | Socioeconomic score (2) | Numerical | Socioeconomic score (2) was determined by Points Business Mapping Ltd | V | V |
|  | Country of origin | Categorical | This feature is relevant only the LightGBM, which handles categorical features |  |  |
|  | Year of immigration | Numerical |  |  |  |
|  | Vaccination rank in the season prior | Numerical | According to the vaccination rank calculation, elaborated in the methods |  | V |
|  | Vaccination rank two seasons prior | Numerical | According to the vaccination rank calculation, elaborated in the methods |  |  |
|  | Vaccination rank three seasons prior | Numerical | According to the vaccination rank calculation, elaborated in the methods |  |  |
|  | Number of respiratory diagnoses in the season prior | Numerical |  |  |  |
|  | Number of respiratory diagnoses two seasons prior | Numerical |  |  |  |
|  | Number of respiratory diagnoses three seasons prior | Numerical |  |  |  |
|  | Cumulative hospitalized days in the season prior | Numerical | Hospitalizations of any reason (were not limited to respiratory infections) |  |  |
|  | Cumulative hospitalized days two seasons prior | Numerical | Hospitalizations of any reason (were not limited to respiratory infections) |  |  |
|  | Cumulative hospitalized days three seasons prior | Numerical | Hospitalizations of any reason (were not limited to respiratory infections) |  |  |
|  | Number of encounters with the healthcare system in the season prior | Numerical | Encounters of any reason (were not limited to respiratory infections) |  |  |
|  | Number of encounters with the healthcare system two seasons prior | Numerical | Encounters of any reason (were not limited to respiratory infections) |  |  |
|  | Number of encounters with the healthcare system three seasons prior | Numerical | Encounters of any reason (were not limited to respiratory infections) |  |  |
|  | Number of prescribed medications in the season prior | Numerical | Medications of any reason (were not limited to respiratory infections) |  |  |
|  | Number of prescribed medications two seasons prior | Numerical | Medications of any reason (were not limited to respiratory infections) |  |  |
|  | Number of prescribed medications three seasons prior | Numerical | Medications of any reason (were not limited to respiratory infections) |  |  |
|  | Chronic illness in the season prior | Binary | A variable that indicates any chronic illness of the chronic diseases described in Table S1 |  |  |
|  | Chronic illness two seasons prior | Binary | A variable that indicates any chronic illness of the chronic diseases described in Table S1 |  |  |
|  | Chronic illness three seasons prior | Binary | A variable that indicates any chronic illness of the chronic diseases described in Table S1 |  |  |
|  | Vaccination proportion at the patient’s clinic, in the season prior | Numerical |  | V | V |
|  | Vaccination proportion at the patient’s clinic, two seasons prior | Numerical |  | V | V |
|  | Vaccination proportion at the patient’s clinic, three seasons prior | Numerical |  | V | V |
|  | Average Vaccination rank of the patient’s family in the season prior | Numerical | According to the vaccination rank calculation, elaborated in the methods. This feature is relevant only for the data set of users with family members within the sample. |  |  |
|  | Average Vaccination rank of the patient’s family two seasons prior | Numerical | According to the vaccination rank calculation, elaborated in the methods. This feature is relevant only for the data set of users with family members within the sample. |  |  |
|  | Average Vaccination rank of the patient’s family three seasons prior | Numerical | According to the vaccination rank calculation, elaborated in the methods. This feature is relevant only for the data set of users with family members within the sample. |  |  |

**Table S4.** Hyper-parameters for the machine learning models

| Algorithm | hyper-parameter | Examined values | Selected value  Basic dataset | Selected value  Family dataset |
| --- | --- | --- | --- | --- |
| Logistic Regression | ‘C’ - Inverse of regularization strength. The regularization aims to limit the magnitude of the models’ parameters in order to avoid overfitting. | ${10}^{-10}, {10}^{-9},{10}^{-8},{10}^{-7},$  ${10}^{-6},{10}^{-5},{10}^{-4},{10}^{-3},$  ${10}^{-2},{10}^{-1},10,{10}^{1}$  $,{10}^{2},{10}^{3},{10}^{4},{10}^{5},{10}^{6},$  ${10}^{7},{10}^{8},{10}^{9}$ | ${10}^{3}$ | ${10}^{-1}$ |
| XGBoost | max_depth - regularization parameter for maximum depth of a tree | 10,15,20 | 10 | 10 |
|  | min_child_weight - the minimum sum of instance weight needed in a child | 1, 3 | 3 | 3 |
|  | colsample_bytree - subsample ratio of columns when constructing each tree | 0.6,0.75 | 0.6 | 0.6 |
|  | colsample_bylevel - subsample ratio of columns for each split, in each level | 0.6,0.75 | 0.75 | 0.75 |
| Light GBM | num_leaves - regularization parameter maximun number of leaves in one tree | 17,31,71,127 | 127 | 127 |
|  | feature_fraction - subsample ratio of columns when constructing each tree | 0.5,0.6,0.75 | 0.5 | 0.5 |
|  | bagging_fraction - subsample ratio of rows when constructing each tree | 0.5,0.6,0.75 | 0.5 | 0.5 |
|  | learning_rate - set to control the weighting of new trees added to the model | 0.005,0.01 | 0.01 | 0.01 |
| Artificial Neural Network | Activation – non-linear activation function of the network | 'relu', 'tanh', 'sigmoid' | 'relu' | 'tanh' |
|  | hidden_layer_sizes – the network architecture given by the number of nodes in the hidden layers. | (5,2), (10,5), (30,10,5), (100,40,10), (100,40,10,5) | (100,40,10,5) | (100, 40, 10) |
|  | learning_rate – learning rate schedule for weight updates | 0.001, 0.01 | 0.001 | 0.001 |
|  | optimizer – optimization algorithm for the backpropagation of the gradients. | ‘lbfgs’, 'Adam' | 'Adam' | 'Adam' |
|  | batch_size - size of minibatches for stochastic optimizers | 32,128,500 | 500 | 128 |

**Table S4.** A detailed evaluation of all predictive models

| Basic dataset | | | | | | | | |
| --- | --- | --- | --- | --- | --- | --- | --- | --- |
|  | **Previous year (PS)** | **Socio demographic model** | **Vaccination decision in the previous season & Sociodemographic model** | **Naïve Bayes** | **Logistic Regression** | **XGBoost** | **LightGBM** | **Neural Network** |
| vALIDATION ROC AUC | - | - | - | 0.87 | 0.9 | 0.91 | 0.91 | 0.91 |
| Train ROC AUC | - | - | - | 0.87 | 0.89 | 0.94 | 0.92 | 0.91 |
| vALIDATION Precision | - | - | - | 0.86 | 0.89 | 0.9 | 0.89 | 0.9 |
| Train Precision | - | - | - | 0.86 | 0.89 | 0.92 | 0.89 | 0.9 |
| vALIDATION Recall | - | - | - | 0.85 | 0.89 | 0.9 | 0.88 | 0.9 |
| Train Recall | - | - | - | 0.85 | 0.89 | 0.92 | 0.88 | 0.9 |
| vALIDATION F1-score | - | - | - | 0.86 | 0.88 | 0.9 | 0.88 | 0.9 |
| Train F1-score | - | - | - | 0.86 | 0.88 | 0.92 | 0.88 | 0.9 |
| TEST ROC AUC | - | - | - | - | - | 0.91 | 0.91 | - |
| TEST Precision | - | - | - | - | - | 0.9 | 0.89 | - |
| TEST Recall | - | - | - | - | - | 0.9 | 0.87 | - |
| TEST F1-score | - | - | - | - | - | 0.9 | 0.88 | - |
| Family dataset | | | | | | | | |
|  | **Previous year (PS)** | **Socio**  **demographic model** | **Vaccination decision in the previous season & Sociodemographic model** | **Naïve Bayes** | **Logistic Regression** | **XGBoost** | **Light GBM** | **Neural Network** |
| vALIDATION ROC AUC | - | - | - | 0.83 | 0.86 | 0.88 | 0.88 | 0.87 |
| Train ROC AUC | - | - | - | 0.82 | 0.86 | 0.96 | 0.9 | 0.88 |
| vALIDATION Precision | - | - | - | 0.87 | 0.89 | 0.9 | 0.89 | 0.89 |
| Train Precision | - | - | - | 0.87 | 0.89 | 0.96 | 0.9 | 0.89 |
| vALIDATION Recall | - | - | - | 0.86 | 0.9 | 0.9 | 0.86 | 0.9 |
| Train Recall | - | - | - | 0.86 | 0.9 | 0.96 | 0.86 | 0.9 |
| vALIDATION F1-score | - | - | - | 0.86 | 0.88 | 0.9 | 0.87 | 0.89 |
| Train F1-score | - | - | - | 0.86 | 0.88 | 0.95 | 0.88 | 0.9 |
| TEST ROC AUC | - | - | - | - | - | 0.88 | 0.88 | - |
| TEST Precision | - | - | - | - | - | 0.9 | 0.89 | - |
| TEST Recall | - | - | - | - | - | 0.91 | 0.86 | - |
| TEST F1-score | - | - | - | - | - | 0.9 | 0.87 | - |
| Basic and family datasets combined | | | | | | | | |
|  | **Previous year (PS)** | **Socio demographic model** | **Vaccination decision in the previous season & Sociodemographic model** | **Naïve Bayes** | **Logistic Regression** | **XGBoost** | **Light GBM** | **Neural Network** |
| vALIDATION ROC AUC | 0.81 | 0.77 | 0.87 | 0.86 | 0.89 | 0.91 | 0.91 | 0.90 |
| Train ROC AUC | 0.81 | 0.8 | 0.89 | 0.86 | 0.89 | 0.94 | 0.91 | 0.90 |
| vALIDATION Positive-Label Precision | 0.7 | 0.64 | 0.76 | 0.6 | 0.8 | 0.8 | 0.62 | 0.8 |
| Train Positive-Label Precision | 0.7 | 0.67 | 0.79 | 0.59 | 0.8 | 0.89 | 0.63 | 0.79 |
| vALIDATION Negative-Label Precision | 0.93 | 0.86 | 0.92 | 0.92 | 0.91 | 0.92 | 0.95 | 0.92 |
| Train Negative-Label Precision | 0.93 | 0.86 | 0.92 | 0.92 | 0.91 | 0.94 | 0.95 | 0.92 |
| vALIDATION Average Precision | 0.89 | 0.82 | 0.89 | 0.86 | 0.89 | 0.9 | 0.89 | 0.9 |
| Train Average Precision | 0.89 | 0.83 | 0.9 | 0.86 | 0.89 | 0.93 | 0.89 | 0.9 |
| vALIDATION Positive-Label Recall | 0.69 | 0.3 | 0.63 | 0.67 | 0.56 | 0.63 | 0.79 | 0.62 |
| Train Positive-Label Recall | 0.69 | 0.32 | 0.65 | 0.67 | 0.55 | 0.71 | 0.79 | 0.62 |
| vALIDATION Negative -Label Recall | 0.93 | 0.96 | 0.96 | 0.9 | 0.97 | 0.96 | 0.89 | 0.96 |
| Train Negative -Label Recall | 0.93 | 0.97 | 0.96 | 0.9 | 0.97 | 0.98 | 0.89 | 0.96 |
| vALIDATION Average Recall | 0.89 | 0.84 | 0.89 | 0.86 | 0.89 | 0.9 | 0.87 | 0.90 |
| Train Average Recall | 0.89 | 0.85 | 0.9 | 0.85 | 0.89 | 0.93 | 0.87 | 0.9 |
| vALIDATION F1-score | 0.89 | 0.81 | 0.89 | 0.86 | 0.88 | 0.9 | 0.88 | 0.9 |
| Train Total F1-score | 0.89 | 0.82 | 0.9 | 0.86 | 0.88 | 0.93 | 0.88 | 0.9 |
| TEST ROC AUC | 0.81 | 0.77 | 0.87 | - | - | 0.91 | 0.91 | - |
| TEST Precision | 0.89 | 0.81 | 0.89 | - | - | 0.9 | 0.89 | - |
| TEST Recall | 0.89 | 0.84 | 0.89 | - | - | 0.9 | 0.87 | - |
| TEST F1-score | 0.89 | 0.81 | 0.89 | - | - | 0.9 | 0.88 | - |

| 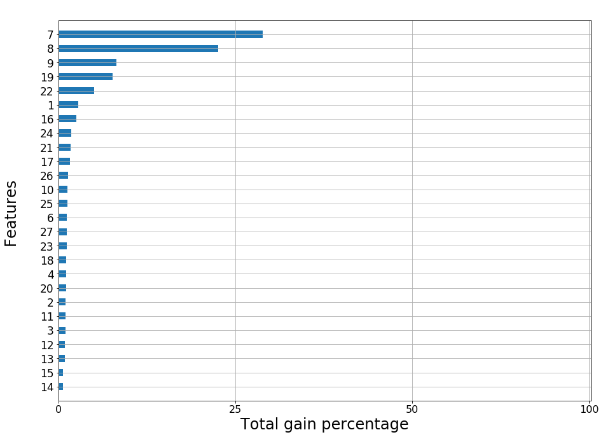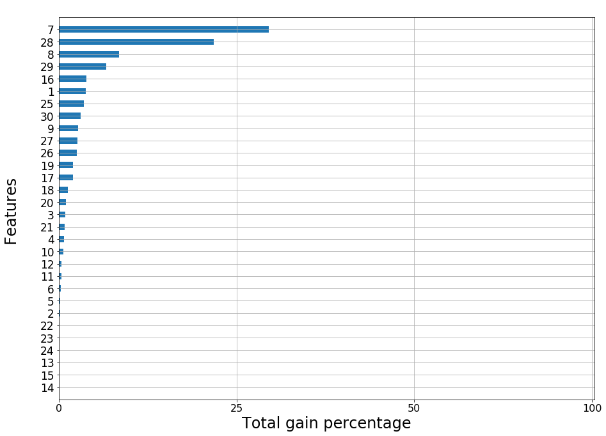 **A**  **B** 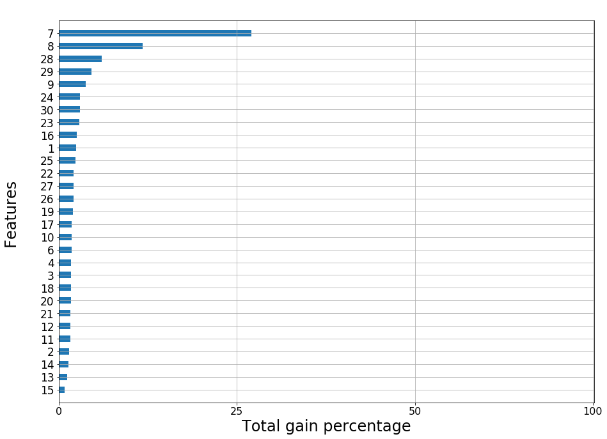 **C** 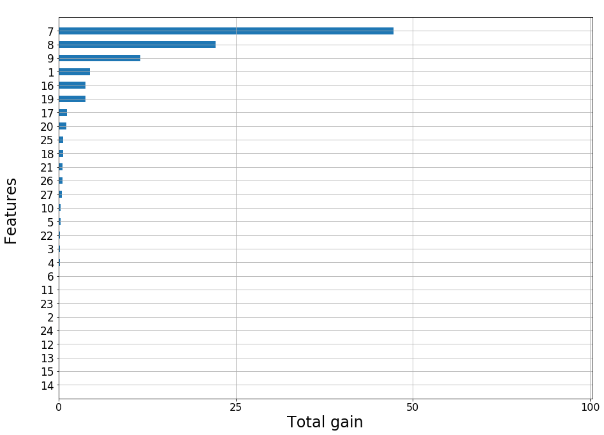 **D** 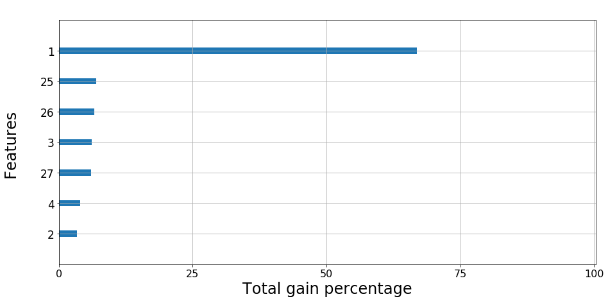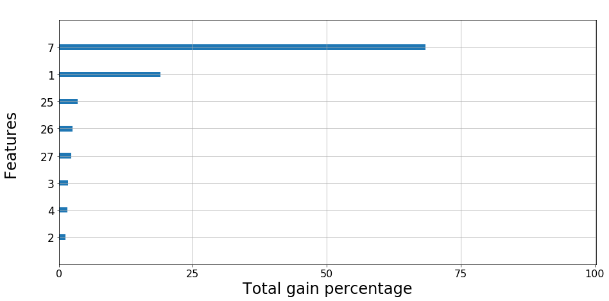 **E**  **F** |
| --- |
| **Figure S2 – Feature importance plots for the following models: (A) XGBoost for the basic dataset (B) LightGBM for the basic dataset (C) XGBoost for the family dataset (D) LightGBM for the family dataset (E) Sociodemographic model (F) Vaccination decision in the previous season & Sociodemographic model**. X-axis values represent the total percentage of information that was gained by the splits of the feature in all the decision trees of the random forest. Y-axis values represent the features’ indices according Table S3 |
